# Clinical factors related to severe enterocolitis after adjuvant CAPOX for colorectal cancer: a retrospective analysis

**DOI:** 10.3332/ecancer.2020.1014

**Published:** 2020-02-24

**Authors:** Tiago Cordeiro Felismino, Victor Hugo Fonseca de Jesus, Bruno Cezar de Mendonça Uchóa Junior, Francisca Giselle Rocha Moura, Rachel P. Riechelmann, Samuel Aguiar Junior, Celso Abdon Lopes de Mello

**Affiliations:** 1 Department of Medical Oncology, AC Camargo Cancer Center, São Paulo, Brazil; 2 Department of Surgical Oncology, AC Camargo Cancer Center, São Paulo, Brazil

**Keywords:** CAPOX, chemotherapy, enterocolitis, colorectal cancer, adjuvant, ARB

## Abstract

**Background:**

CAPOX regimen is a standard option in stage III adjuvant colon cancer. Gastrointestinal toxicity is well described with fluoropyrimidine regimens and can be life-threatening. Identification of risk factors associated with severe gastrointestinal toxicity may help clinicians when choosing the adjuvant regimen.

**Materials and Methods:**

We retrospectively analysed 61 patients treated with adjuvant CAPOX. Our primary objective was to estimate the incidence of severe chemotherapy-induced enterocolitis among patients treated with CAPOX. A secondary objective was to describe the main demographic and clinical characteristics of these patients. A univariate logistic regression was performed to estimate the odds ratio (OR) with a 95% CI to identify a predictor for severe enterocolitis.

**Results:**

Grade 3 diarrhoea was reported in 10 patients (16.3%). Admissions to hospital due to toxicity occurred in nine cases. Reasons for hospitalisation were severe enterocolitis in eight cases (13.1%) and rectal bleeding plus thrombocytopenia in one case. Age > 70 years (OR 9.6; 95% CI 1.81–50.6; *p* = 0.008), primary surgery involving right/transverse colon (OR 16.8; 95% CI 2.88–98.8; *p* = 0.002) and Angiotensin II Receptor Blocker (ARB) use (OR 8.14; 95% CI 1.64–40.3; *p* = 0.010) were associated with severe enterocolitis.

**Conclusion:**

Our data showed that adjuvant CAPOX induced severe enterocolitis in 13.1% of patients. In addition, we found that advanced age, right colectomy and concurrent use of ARB were statistically associated with these events. Awareness of these factors could be easily incorporated into the treatment decision and patient orientation.

## Introduction

Adjuvant chemotherapy with fluoropyrimidine plus oxaliplatin is the mainstream treatment for stage III, resected colorectal adenocarcinoma [1]. FOLFOX, FLOX and CAPOX are acceptable regimens based on phase 3 trial data [2–4]. However, there are no randomised studies comparing these regimens in the adjuvant setting. Treatment choice is based on physician’s preferences, reimbursement, infusion-pump and long-term catheters availability, and toxicity profile.

Recently published, the IDEA study combined analysis [5] compared 3 versus 6 months of oxaliplatin-based therapy (FOLFOX or CAPOX) in stage III colon cancer patients. The primary endpoint of non-inferiority 3y-DFS was not met. However, the clinical relevance of this difference is questionable. Indeed, 3y-DFS was of 74.6% for 3 months versus 75.5% for 6 months of therapy (HR 1.07, 95% CI 1.00–1.15). Neuropathy was significantly decreased in the 3-month treatment group. Interestingly, although not pre-specified, the chemotherapy regimen might have affected the outcomes.

Gastrointestinal toxicity is well described for fluoropyrimidine-based regimens [6]. Diarrhoea and oral mucositis are the typical adverse events of this class of drugs. The severity of toxicity is influenced by many clinical and pharmacological factors, including the mode of drug administration (continuous versus bolus). Capecitabine and infusional 5-FU induce less diarrhoea as compared to bolus 5-FU [7, 8], for example.

Any grade of diarrhoea is commonly observed for the combination of capecitabine plus oxaliplatin [9]. However, severe gastrointestinal toxicity is not common, but it can be life-threatening if not adequately recognised and treated. Enterocolitis is characterised by diarrhoea and small and large bowel wall alterations. This condition, in general, is not associated with neutropenia and has an early onset after treatment start. It was well described in patients treated with FLOX regimen (bolus 5-FU plus oxaliplatin) in the adjuvant setting [10].

In addition, comorbidities and concurrent medication use are highly frequent in the colorectal cancer population. As a result, drug interaction is a major problem and it is usually neglected in patients on chemotherapy. In the general population, Angiotensin II Receptor Blockers (ARBs) are frequently used for blood-pressure control. Olmesartan has been linked to a very rare enteropathic syndrome characterised by chronic diarrhoea and eventually to weight loss and malnutrition [11]. One possible mechanism is the inhibition of TGF-beta, an important mediator of the gut homeostasis [12].

After the results of the IDEA trial favouring CAPOX for most of the patients, our centre gradually changed the recommendation of adjuvant FOLFOX to 3 months of CAPOX, particularly in low-risk stage III (T1–T3, N1) patients. A higher incidence of severe diarrhoea and enterocolitis demanding hospitalisation was observed in this period. Here, we report our data regarding adjuvant CAPOX focusing on the incidence of severe chemotherapy-induced enterocolitis and the clinical characteristics of patients who experienced this toxicity.

## Methods

This is a retrospective analysis of patients treated at the AC Camargo Cancer Center in Brazil between November 2016 and September 2018. The study was initiated after the Institutional Ethics Committee’s approval.

We conducted an analysis of consecutive patients referred to the Department of Medical Oncology with the inclusion criteria: age > 18 years, diagnosis of colon or rectum adenocarcinoma, curative-intent surgery, pathologic TNM stage II or III, creatinine clearance > 50 mL/min, and who had received adjuvant CAPOX (Capecitabine plus Oxaliplatin). Our primary objective was to estimate the incidence of severe chemotherapy-induced enterocolitis among patients treated with CAPOX. A secondary objective was to describe the main demographic and clinical characteristics of these patients. Severe chemotherapy-induced enterocolitis was defined as diarrhoea grade >3 that resulted in hospital admission with any of the following: antibiotics use, sepsis, imaging finding of large or small bowel thickening, distension and obstruction. Descriptive statistics were used for demographics and main results. Data were collected from medical files.

A univariate logistic regression was performed to estimate the odds ratio (OR) with a 95% CI to identify predictor for severe enterocolitis. Variables included were age (<70 years versus >70 years), sex, Eastern Cooperative Oncology Group (ECOG) performance status (0 versus 1 and 2), type of primary tumour surgery (involving right and transverse colon versus not involving right and transverse colon), postoperative antibiotics use, concurrent ARBs use, capecitabine dose (<2,000 mg/m^2^ versus 2,000 mg/m^2^) and oxaliplatin dose (<130 mg/m^2^ versus 130 mg/m^2^) at first cycle. Differences with a two-sided *p* < 0.05 were considered statistically significant.

## Results

Between November 2016 and September 2018, 61 patients received adjuvant CAPOX after curative-intent surgery for stage III colorectal adenocarcinoma. Table 1 shows the clinical and demographic characteristics of the study population. The median age was 57 years (range: 28–77 years). Nine patients (14.8%) were older than 70 years. Thirty-four patients (55.7%) were females, and ECOG 0 or 1 was found in 93.5% of patients. Median follow-up time was 8.96 months (95% CI: 7.18–10.75). Sidedness distribution was: right and transverse colon tumour in 19.6% and left colon and rectum in 77%.

The most frequent type of primary tumour surgery was rectosigmoidectomy (39 patients; 63.9%), followed by right colectomy in (12 patients; 19.6%). Two patients underwent neoadjuvant chemoradiotherapy for rectal adenocarcinoma. Data for postoperative hospitalisation length were available in 47 cases; median days in hospital was 4 (range: 2–31 days). After surgery, protective ileostomy and colostomy were performed in 11 (18%) and 2 (3.3%) patients, respectively. Sixty patients had pathological stage III and one had stage II colorectal cancer according to the AJCC eighth edition. Seven patients received postoperative antibiotics (mainly ceftriaxone plus metronidazole).

Regarding adjuvant CAPOX, median prescribed capecitabine and oxaliplatin doses were 2,000 mg/m^2^ (range: 1,600–2,000 mg/m^2^) and 130 mg/m^2^ (range: 100–130 mg/m^2^), respectively. Number of prescribed cycles were 4, 6 and 8 in 44 (72.1%), 1 (1.6%) and 16 (26.2%) patients, respectively. The median time between surgery and the first chemotherapy cycle was 49 days (95% CI: 43.5–54.4). Among all patients that started adjuvant therapy, 23 (37.7%) did not complete all intended cycles.

Grade 3 diarrhoea was reported in ten patients (16.3%). Hospital admissions due to general toxicity occurred in nine cases (14.7%). Eight out of nine (88.9%) hospitalisations were due to enterocolitis and one due to rectal bleeding plus thrombocytopenia.

**Figure 1.**
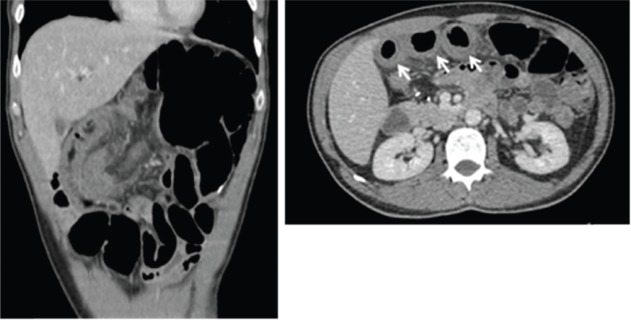
CT scan showing bowel thickening and dilation. Arrow shows small bowel thickening.

Regarding patients who were hospitalised due to severe enterocolitis (*N* = 8), the median age was 68.5 years (range: 50–77 years), four (50%) were older than 70 years (Table 2). Five patients were females, and seven patients had baseline performance status ECOG 0. Primary surgery was right colectomy in six (75%) and rectosigmoidectomy in two (25%). No patient had a protective ostomy. The median time between surgery and the first cycle was 45 days (95% CI: 42.4–47.5). Six patients were admitted after cycles 1 (3 pts) and 2 (3 pts), one patient after cycle 3 and one after cycle 5. The median length of hospitalisation was 15 days (range: 8–119 days). None of the patients presented with severe myelotoxicity or alopecia upon admission. All patients received antibiotics, mainly quinolones and metronidazole. Three patients were admitted to the intensive-care unit. No deaths occurred. During admission, seven patients underwent abdominal computed tomography (CT) scan. The main findings were wall thickening, dilated bowel and air-fluid levels, as shown in Figure 1. The most frequent segment affected was the ileum. After discharge from the hospital, three patients were switched to FOLFOX and one patient re-started CAPOX.

Data regarding concurrent medications were collected from medical files. Among patients who were hospitalised for enterocolitis, main findings were: five (62.5%) reported the use of Losartan 50–100 mg/d (ARB) for blood-pressure control, two reported the use of Sinvastatin 10 mg/day, one used folic acid (5 mg/day), one used metformin (850 mg/day) and one enalapril (10 mg/day)

A univariate analysis (Table 3) was performed for each variable in this retrospective cohort. Age > 70 years (OR 9.6; 95% CI 1.81–50.6; *p* = 0.008), primary surgery involving right/transverse colon (OR 16.8; 95% CI 2.88–98.8; *p* = 0.002) and ARB use (OR 8.14; 95% CI 1.64–40.3; *p* = 0.010) were associated with severe enterocolitis. Gender (*p =* 0.68), ECOG status (*p* = 0.67), postoperative antibiotics (*p* = 0.94), capecitabine dose (*p* = 0.58) and oxaliplatin dose (*p* = 0.78) were not associated with severe enterocolitis.

## Discussion

The IDEA trial induced a shift in the clinical practice of adjuvant therapy for stage III colon cancer was observed. Three months of CAPOX is replacing the 6 months of adjuvant FOLFOX for a considerable number of patients. More recent data also show a possible reduction in the length of adjuvant treatment in high-risk stage II patients [13]. Here, we describe the characteristics of eight patients who presented life-threatening enterocolitis after adjuvant CAPOX. Age, right colectomy and the use of ARB were more frequent in this group of patients.

Diarrhoea is a well-described side effect of the CAPOX regimen. However, the rate of grade 3 and 4 diarrhoea varies among the trials. A systematic review compared CAPOX versus FOLFOX in the first-line setting in metastatic colorectal adenocarcinoma [14]. There were no differences in overall efficacy endpoints; however, grade 3/4 diarrhoea was more incident in the CAPOX treated patients (OR = 1.76, 95% CI 1.43–2.16, *p* < 0.00001). A retrospective study of a cohort from Canada [15] compared 394 patients treated with adjuvant FOLFOX or CAPOX in stage III colon cancer. CAPOX was associated with diarrhoea (31.8% versus 9.0%, *p* < 0.0001) and hand-foot syndrome (19.9% versus 2.1%, *p* < 0.0001). In the IDEA [5] and XELOXA [16] trials, the frequency of grade 3/4 diarrhoea was 8.8% (for 6 months) and 19%, respectively. In our study, grade > 3 diarrhoea occurred in 16% of patients.

The mechanism behind GI toxicity with the oxaliplatin-based regimen is not totally clarified. Protracted infusion 5FU or capecitabine is believed to cause less mucosal destruction and consequently less diarrhoea. In the XELOXA trial [16], stage III patients received the combination of CAPOX or routine bolus 5-FU (Mayo Clinic or Roswell Park Regimens). Grade 3/4 diarrhoea was observed in 19%, 16% and 29% of patients treated with CAPOX, Mayo and Roswell Park protocol, respectively, with median time to symptom onset of 36, 36 and 34 days, respectively.

In addition, the pattern and severity of gastrointestinal toxicity were distinct between the bolus and infusional 5FU studies. It is noteworthy that in the NSABP-C07 trial, a high rate of a specific condition named enteropathy syndrome (ES) was observed in 4.3% of patients [10]. This syndrome was described by the presence of severe diarrhoea and bowel wall injury (BWI) in the small and large intestine with endoscopic or radiographic evidence of bowel wall thickening or ulceration. Patient treated with FLOX regimen had a nearly double chance of developing ES as compared to patients treated with bolus 5-FU and Leucovorin (64.6% × 35.4%, respectively, *p* < 0.01). On the contrary, no ES was described in the FOLFOX arm of the MOSAIC trial, despite some controversies about the characterisation of the syndrome in this trial. Here, in our analysis, 13% of patients presented the enteropathy syndrome with diarrhoea and tomographic abnormalities and most occurred after the first and second cycles. Indeed, the enteric syndrome is not related to the cumulative dose of chemotherapy. It is early-onset as shown by previous studies and our data. Neither diarrhoea seems to be related to prolonged exposure to CAPOX. By analysing the IDEA trial [5] data, we could confirm that grade 3/4 diarrhoea induced by CAPOX was very similar comparing 3 versus 6 months (7.4% versus 8.8%, respectively).

Elderly patients are more likely to experience toxicity from fluoropyrimidine-based regimens [17]. Our data showed that toxicity was more frequent in older patients (>70 years). The literature shows conflicting results regarding the overall survival impact of oxaliplatin for older patients in the adjuvant scenario [18]. On the other hand, oxaliplatin-based adjuvant chemotherapy seems more toxic in the elderly. A pooled analysis of prospective adjuvant trials in stage III colon cancer evaluated the incorporation of oxaliplatin [19] and showed more grade 3/4 adverse events among patients older than 70y treated with oxaliplatin (52% versus 35%). Grade 3/4 diarrhoea was also more frequent in the oxaliplatin-based arms (20% versus 16%). The efficacy and safety of capecitabine plus bevacizumab was evaluated in the AVEX trial [20] and grade 3 or higher gastrointestinal toxicity was observed only in 7% of patients, showing that the combination with oxaliplatin considerably increases the GI toxicity.

Another important factor that was associated with increased gastrointestinal toxicity was right colon tumour. Among patients admitted for enterocolitis, six (75%) had a surgery involving right/transverse colon. Currently, it is clear that right colon carcinoma has worse prognosis as compared to left sided tumours [21]. Recent data have demonstrated that right side colon has a complex layer composed of microorganism that covers the intestinal mucosa. This biofilm is almost absent in the left colon [22]. This is just one important difference between these two parts of the large bowel. One potential explanation for the presence of more events in patients undergoing right colectomy could be the impairment of the microbioma that is responsible for the intestinal homeostasis and eventually mucosal integrity and resistance to drug injury, such as chemotherapy.

Early recognition and treatment of life-threatening toxicity due to chemotherapy is mandatory to prevent fatal events. In the context of severe adverse event and fluoropyrimidine use, DPD deficiency should be suspect. However, it is known that the majority of grades 3–5 toxicities after 5FU and capecitabine exposure are not due to this enzyme deficiency. Baseline screening of patients undergoing first treatment with this class of drug is not recommended by any guideline due to low accuracy of the test and the costs involved. However, it is important to continue searching for molecular and non-molecular conditions that could be related to severe GI toxicity.

In our study, with a limited sample of patients, we found a statistically significant association in the univariate analysis between enterocolitis and the concurrent use of ARB (mainly Losartan), that is commonly prescribed to control blood pressure in Brazil. In our cohort, among patients admitted with enterocolitis, 62.5% reported the use of losartan and it translated to eightfold increase in developing this event. (OR of 8.14). Other potential medications that could impair the microbiota equilibrium such as antibiotics, metformin and even statins were not associated with increased chance of developing severe enterocolitis, but we have to remind that due to small sample size a multivariate analysis could not be carried out and confidence intervals are wide.

A putative mechanism linking ARB to gastrointestinal toxicity after CAPOX could be interaction of this drug with transforming growth factor beta (TGF-β) pathway. Preclinical data have demonstrated that losartan suppresses active transforming growth factor levels via an angiotensin II-mediated down-regulation of TGF-β1 [23]. In its turn, TGF-β plays an important role in the gut mucosa. Inhibition of the TGF-β signalling disrupts gut homeostasis by promoting gut inflammation and possibly enterocolitis [24]. A sprue-like enteropathy related to olmesartan has been described by many series [25], and case reports with other ARBs such as losartan and valsartan have described the association of these agents with chronic diarrhoea and weight loss [25–27]. The gastrointestinal symptoms are late-onset, usually after many months of use. We hypothesised that synergistic action of enterotoxic agents such as capecitabine plus oxaliplatin and losartan could trigger a major damage to the intestinal mucosa with inflammatory diarrhoea due to the crucial role of TGF-β in the in the gut immune homeostasis.

## Conclusion

In summary, our retrospective data showed that adjuvant CAPOX for stage III colorectal cancer induced severe enterocolitis in 13.1% of patients. All of these patients were admitted and most of them presented similar findings such as long hospital stay and radiological findings of enterocolitis. One important finding of our study is that age, right colectomy and concurrent use of losartan were associated with these events in the univariate analysis. Since CAPOX has been more widely used in the context of post-operative treatment, awareness of factors associated with severe and life-threatening toxicity would help physicians to early diagnosis of severe enterocolitis and better guide adjuvant therapy. Our data must be validated by larger studies.

## Conflicts of interest

None.

## Funding

This research did not receive any specific grant from funding agencies in the public, commercial or not-for-profit sectors.

## Figures and Tables

**Table 1.** Demographics.

	N = 61
Med age	57 (28–77)
> 70 years	9 (14.8%)
Gender	
Female	34 (55.7%)
Male	27 (44.3.%)
ECOG	
0	53 (86.9%)
1	4 (6.6%)
2	1 (1.6%)
Unknown	3 (4.9%)
Surgery	
Right Colect	12 (19.6%)
Tranverse Colect	1 (1.6%)
Left Colect	7 (11.5%)
Retossigmoidectomy	39 (63.9%)
APR (rectal)	1 (1.6%)
Total colectomy	1 (1.6%)
Ostomy	
Yes	13 (21.3%)
No	45 (73.8%)
Unknown	3 (4.9%)
ATB after surgery	
No	45 (73.7%)
Yes	7 (11.4%)
Unknown	9 (4.9%)
N CAPOX cycles prescribed	
4	44 (72.1%)
6	1 (1.6%)
8	16 (26.2%)
ARB use	
Yes	14 (23.0%)
No	47 (77.0%)

ARB: Angiotensin II Receptor Blocker, colect: colectomy.

**Table 2.** Patients admitted with severe enterocolitis.

Pt	Gender	Age	Surgery	Staging	Use of ARB	Protect ostomy	Cycle	Antibiotics for Enterocolitis
#1	M	53	Right Colectomy	pT3pN1	No	No	1st	Cefepime
#2	F	51	Right Colectomy	pT3pN1	No	No	2nd	Cefepime + Metronidazole
#3	F	65	Rectosigmoidectomy	pT3pN1	Yes	No	3th	Ceftriaxone + Metronidazole
#4	F	73	Right Colectomy	pT3pN1	Yes	No	1st	Piperacilin-Tazobactam -> Meropenem + Vancomycin
#5	F	73	Right Colectomy	pT3pN1	No	No	5th	Ciprofloxacin + Metronidazole
#6	F	78	Right Colectomy	pT3pN1	Yes	No	2nd	Ciprofloxacin + Metronidazole
#7	M	73	Right Colectomy	pT3pN2	Yes	No	1st	Piperacilin-Tazobactam -> Vancomycin ->Meropenem
#8	M	62	Rectosigmoidectomy	pT4pN1	Yes	No	2nd	Piperacilin-Tazobactam

Pt: Patient; ARB: Angiotensin II Receptor Blocker.

**Table 3.** Univariate analysis.

		Enterocolitis (%)	OR	95%CI	p
Age	< 70 years > 70 years	7.7% 44.4%	1 9.60	1.81–50.6	**0.013**
Colectomy	Non right / transverse Right / transverse	4.3% 42.9%	1 16.8	2.88–98.8	**0.002**
ARB use	No Yes	6.4% 35.7%	1 8.14	1.64–40.3	**0.010**
Gender	Male Female	11.1% 14.7%	1 1.37	0.29–6.37	0.68
ECOG	0 1 and 2	13.2% 20%	1 1.64	0.16–16.90	0.67
Antibiotics use after surgery	No Yes	13.3% 14.3%	1 1.08	0.11–10.6	0.94
Capecitabine dose	< 2000mg/m^2^ 2000mg/m^2^	8.3% 14.3%	1 1.83	0.20–16.51	0.58
Oxapliatin dose	< 130mg/m^2^ 130mg/m^2^	16.7% 12.7%	1 0.72	0.74–7.19	0.78

ARB: Angiotensin II Receptor Blocker.
